# Fermentation Technologies for the Optimization of Marine Microbial Exopolysaccharide Production

**DOI:** 10.3390/md12053005

**Published:** 2014-05-22

**Authors:** Ilaria Finore, Paola Di Donato, Vincenza Mastascusa, Barbara Nicolaus, Annarita Poli

**Affiliations:** 1 Consiglio Nazionale delle Ricerche (C.N.R.), Institute of Biomolecular Chemistry, Via Campi Flegrei 34, 80078 Pozzuoli, NA, Italy; E-Mails: ilaria.finore@icb.cnr.it (I.F.); paola.didonato@icb.cnr.it (P.D.D.); vincenza.mastascusa@uniparthenope.it (V.M.); bnicolaus@icb.cnr.it (B.N.); 2 Department of Sciences and Technology, University of Naples, “Parthenope” Centro Direzionale Napoli (Isola C4), 80143 Naples, Italy

**Keywords:** exopolysaccharides, extremophiles, fermentation, marine microorganisms, statistical tool

## Abstract

In the last decades, research has focused on the capabilities of microbes to secrete exopolysaccharides (EPS), because these polymers differ from the commercial ones derived essentially from plants or algae in their numerous valuable qualities. These biopolymers have emerged as new polymeric materials with novel and unique physical characteristics that have found extensive applications. In marine microorganisms the produced EPS provide an instrument to survive in adverse conditions: They are found to envelope the cells by allowing the entrapment of nutrients or the adhesion to solid substrates. Even if the processes of synthesis and release of exopolysaccharides request high-energy investments for the bacterium, these biopolymers permit resistance under extreme environmental conditions. Marine bacteria like *Bacillus*, *Halomonas*, *Planococcus*, *Enterobacter*, *Alteromonas*, *Pseudoalteromonas*, *Vibrio*, *Rhodococcus*, *Zoogloea* but also Archaea as *Haloferax* and *Thermococcus* are here described as EPS producers underlining biopolymer hyperproduction, related fermentation strategies including the effects of the chemical composition of the media, the physical parameters of the growth conditions and the genetic and predicted experimental design tools.

## 1. Introduction

The aquatic habitats represented by the oceans, seas and lakes are the widest sources of biological and chemical diversity that not only constitute a productive reserve of unexploited and/or unknown microorganisms [1,2,3] but are also a great resource of new biomolecules displaying potential biotechnological applications in pharmaceutical, medical [4], cosmetic [5], feed, food and enzyme industries [6]. The singleness of these new microorganisms makes them ideal candidates as models to study the ecosystem apart from providing opportunities to discover new compounds of commercial importance. Among the microbial products, exopolysaccharides (EPS) possess significant main physiological functions and various practical applications. During the last years, the increased demand for new and natural polymeric materials by several industrial fields, such as pharmaceutical, food and others, has moved the interest to the polysaccharides produced by microorganisms. Moreover, EPSs find applications in environmental biotechnology where they are employed in soil and water bioremediation, decontamination and detoxification processes [7,8]. These polymers differ from others that are derived from plants or algae, for numerous valuable qualities. Although the world market is dominated by polysaccharides derived from plants and marine macroalgae, there are many microorganisms (Gram-positive and Gram-negative Bacteria, Archaea, Fungi and some microscopic Algae) that are able to produce polysaccharides as extracellular polymers [9]. Bacterial EPSs that are widely used in food, pharmaceutical and chemical industries as bioflocculants, bioabsorbents, drug delivery agents, *etc*. are produced by *Xanthomonas*, *Leuconostoc*, *Pseudomonas*, *Alcaligenes* genera, which synthesize xanthan, dextran, gellan, curdlan. Despite the wide diversity of microbial EPS scenario, xanthan and gellan are the only two EPSs of microbial origin that are used as additives in the food industry in the United States and Europe [10].

Although diverse microbial exopolysaccharides (EPS) have been studied during the last years, little information is available regarding their hyperproduction. In this review, we describe some microbial EPS producers and the related fermentation strategies and genetic tools adopted in order to enhance the EPS yields. We also go through the features of the EPS production process which can vary in order to increase the yield of polymer. Several factors can affect this complex process: For instance, the medium composition and the fermentation conditions (temperature, pH, aeration, *etc*.) represent variables that act to improve the polymer release. Moreover, since this process is genetically determined, several studies and strategies are being developed to intervene at the genome level with the aim to boost EPS productivity. 

## 2. General Information of Microbial Exopolysaccharides: Chemical Structure, Ecological Role and Biosynthesis

EPSs are divided into two main groups, homopolysaccharides and heteropolysaccharides. The first are made up of a single type of monosaccharide: This is the case, for example, for levan or dextran. Heteropolysaccharides containing three or four different monosaccharides display a backbone structure made up of a repeating unit constituted by ten or less monomers [11]. The most common units constituting the EPSs from marine microorganisms are d-arabinose, d-ribose and d-xylose as pentoses; d-glucose, d-galactose, d-mannose, d-allose, l-rhamnose and l-fucose are found as hexoses, while d-glucosamine, d-galactosamine, d-glucuronic acids and d-galacturonic acids are found as amino sugars and uronic acids, respectively. Other organic or inorganic substituents, such as phosphate, sulfate, succinic, acetic and pyruvic acids can also be found [12]. Several are the examples of polymers with a linear structure and a molecular weight between 1 × 10^5^ to 3 × 10^5^ Da [13]. Some EPSs are neutral polymers but many of them are polyanionic because of the presence of ketal-linked pyruvate or inorganic residues such as phosphate or sulfate or uronic acids. Moreover, the 1,4-β- or 1,3-β- linkages between monosaccharides are most commonly found in the rigid backbone of EPSs while the 1,2-α- or 1,6-α- linkages are typical for the more flexible ones [14].

EPSs are produced in response to biotic stress (e.g., competition), abiotic stress factors (e.g., temperature, light intensity, pH, salinity) and/or as a strategy of adaptation to an extreme environment like in the case of acidophilic or thermophilic species belonging to the Bacteria and Archaea domains [10]. The physiological role of EPS depends on the natural environment in which the microorganisms live. Most of the functions ascribed to EPSs are of protective nature. They could support the microorganisms to tolerate extreme conditions such as extremely high or low temperatures, salinity and limited availability of nutrients by surrounding the cells and their immediate environment [11]. In addition, they are involved in various cell functions such as adhesion to surfaces and to other organisms, in biofilm production, as a support in pathogenic and virulence mechanisms or as storage of reserve carbon sources. 

EPS biosynthesis includes three main phases: assimilation of a carbon substrate, intracellular synthesis of the polysaccharide and its exudation out of the cell [15]. Although the exopolysaccharide production is a process that involves a noticeable energy cost, due to the strong need of carbon input for the microorganisms, the gain coupled with their presence is considerably higher compared to the costs (considering the growth increase and the survival of the microbial producers) [16,17]. The mechanism of EPS biosynthesis depends on the type of polysaccharide. In the case of homopolysaccharides (dextran, levan and mutan, *etc*.), the polymerization reaction occurs thanks to an extracellular glycosyltransferase that is able to transfer a monosaccharide unit from a disaccharide by increasing the length of the polysaccharide chain. In the case of heteropolysaccharides with repeating units, the assembly of repeating units takes place in the cytoplasm through the action of specific glycosyltransferases responsible for the continuous transfer of sugar residues to a lipophilic carrier. Once formed, the sugar chain is transferred outside the cells and polymerized. The extent of the release of the polymers and their eventual chemical modification, *i.e.*, acetylation or pyruvylation reactions, and the addition of phosphate or sulfate substituents are under enzymatic and genetic control. For instance, the knowledge of the glycosyltransferase gene map has been useful in predicting the structure of the repeating unit of EPS in lactic acid bacteria [18,19,20]. Since the microbial EPS production is related to a specific gene cluster, the knowledge of the genome sequence of EPS producers is surely the crucial point for the optimization of their biosynthesis through a system biology approach [21,22]. In addition, the manipulation of genes responsible for the EPS biosynthesis could interfere with the chemical-physical characteristics of EPSs and finally with the properties and potential application of these polymers. However, the regulation of EPS biosynthesis, overall in marine EPS producers, is still very poorly understood: More knowledge on the genetics and biochemistry of EPS biosynthesis is needed in order to engineer polysaccharide properties by modifying production, composition and chain length [23].

## 3. Marine EPS Producers: Conventional and Non-Conventional (Extremophile) Microorganisms

The marine habitats represent a vast source of natural products, and their microbial inhabitants have developed unique metabolic and physiological capabilities to produce novel metabolites [6,24]. Although more than 10,000 metabolites with biological activities have been isolated from marine microorganisms, most of the marine microbial world remains unexplored [6].

Kumar *et al.* [9] isolated the species *Planococcus maitriensis* Anita I from the coastal sea water area of Bhavnagar (India). This microorganism was able to produce an EPS possessing an oil spreading potential comparable to Triton X100 and Tween 80, thus resulting in being suitable for bioremediation, enhanced oil recovery and cosmetic applications.

Iyer *et al*. [25] described an acidic EPS with a high content of uronic acid, named 71a, produced by *Enterobacter cloacae* collected from marine sediments in India. The EPS 71a showed emulsifying properties comparable to commercial gums. Mancuso *et al.* [2,26] described the effect of growth temperatures on the EPS production by the strains CAM025 and CAM036, two marine bacteria isolated from sea ice and seawater sampled in the Southern Ocean (Antarctica) and belonging to the genus *Pseudoalteromonas*. 

A branched EPS that has gelling properties was produced by the *Pseudoalteromonas* strain HYD 721 isolated from a deep-sea hydrothermal vent of the East Pacific Rise [27], while an EPS with a high degree of acetylation that showed flocculation behavior and a bio-sorption capacity was produced by the *Pseudoalteromonas* strain SM9913 isolated from deep-sea sediments in the gulf of the Yellow Sea (China) [28,29]. The structure of a highly complex alpha-mannan polymer and its ecological role as crioprotector of a novel EPS from the arctic sea ice bacterium *Pseudoalteromonas* sp. strain SM20310 was described by Liu *et al*. [30].

Other cases of microorganisms with an appreciable capability to produce EPS include Gamma-Proteobacteria, species belonging to *Idiomarina* and *Alteromonas* genera, in particular *Alteromonas hispanica* and also species belonging to the Alpha-Proteobacteria group, like *Salipiger mucosus* and *Palleronia marisminoris* [31]. Vincent *et al.* [32] described the isolation of *Alteromonas* sp. from the epidermis of a polychaete annelid, *Alvinella pompejana*, living in the deep sea hydrothermal vents of the East Pacific Rise, able to produce a heteropolysaccharide with high levels of uronic acids and pyruvate, and showing anticoagulant and bone healing properties [33,34,35]. Bozzi *et al*. [36,37] described an EPS capable of binding heavy metals produced by the *Alteromonas* strain 1644 isolated from *Alvinellidae* collected from the East Pacific Rise. The marine bacterium *Pseudomonas* sp. strain S9 was found to produce EPS during both growth and total energy source and nutrient starvation [38]. Lim *et al*. [39] described the physicochemical properties of some EPSs (capsular and water soluble polysaccharides) produced by the marine bacterium *Zoogloea* sp. KCCM10036: These EPSs showed an excellent flocculating activity. Exopolymer-producing halophilic luminescent bacteria were isolated from water samples collected from sampling sites of the rivers Mandovi and Zuari estuarine network connecting the coastal regions of Goa [40]. Among the screened isolates, the halophilic luminescent *Vibrio harveyi* strain VB23 showed the ability to form a viscous exopolymer possessing a good emulsifying activity.

The physicochemical properties of bioadhesives produced by a marine biofouling bacterium, *Vibrio alginolyticus*, were described by Muralidharan and Jayachandran [41]. According to gas chromatography/mass spectrometry (GC/MS) analysis, this EPS was formed by glucose, xylose, aminoribose and aminoarabinose with a molar ratio of 2:1:9:1. Indeed, the exopolysaccharide released by *V. fischeri* was composed by glucose, mannose, galactose, rhamnose and arabinose with a molar ratio of 3.4:3.7:1.5:0.6:0.7 [42].

*Shewanella colwelliana*, a marine microorganism that lives in association with oysters, synthesized an atypical EPS involved with the irreversible adhesion process of the bacterium to substrates. Furthermore, the production of the exopolymer was improved by growing this bacterium on marine agar. The purified exopolymer showed the presence of Ca, S, P and Si (40%–45%), carbohydrates (15%–35%), lipids (10%), and proteins (<5%) with glucose being the most abundant component of the carbohydrate moiety [43]. 

A potential hydrocarbon tolerance of *Rhodococcus erythropolis* PR4 was ascribed to two acidic EPSs (FR1 and FR2) produced by this marine bacterium. In particular FR2 was found to be composed of d-galactose, d-glucose, d-mannose, pyruvic acid and d-glucuronic acid, and also contained stearic and palmitic acids attached via ester bonds [44] to the sugar backbone.

Recently, several extremophilic microorganisms isolated from extreme environments (deep sea hydrothermal vents, Arctic and Antarctic ecosystems, saline lakes, geothermal springs, *etc.*), have been studied as potential sources of valuable EPSs in attempt to find new biopolymers with unique properties [3,23]. Since extremophiles offer important insights into biology and evolution studies, they could be considered as a valuable resource for exploitation in novel biotechnological processes. During the last decades, several reports have stated the production of EPSs produced by extremophilic microorganisms. These EPSs have been deeply investigated because of their widely diversified structure, biosynthesis, composition and functional properties even if only few of them have been used for industrial scopes. Nevertheless, the extremophilic microorganisms and their exopolysaccharides could find various biotechnological applications, thanks to some advantageous features like for example a short duration of fermentative processes in the case of thermophilic bacteria or an effortless production of stable emulsions of EPSs in the case of psychrophiles [26]. 

In the Archaea Domain a valuable source of EPS is embodied by bacteria isolated from salt lakes, volcanic and hydrothermal marine areas, like for example species of the genera *Methanosarcina*, *Haloferax*, *Haloarcula*, *Thermococcus* and *Sulfolobus*; *Thermococcus litoralis* and *Archaeoglobus fulgidus* in particular produced a peculiar biofilm composed of a consortium of cells immobilized inside an exopolymer, which may contribute to concentrating nutrients [45]. It is noteworthy that the first reported exopolymer was secreted by the halophilic archaebacterium *Haloferax mediterranei*, which was isolated from the Mediterrean Sea [46,47]. During the fermentation process in static conditions, the cells were found to be enveloped by an amorphous matrix. Notwithstanding, the growth conditions seemed to not affect the production of the polymer, and the extent of the cell aggregation in unshaken cultures appeared to be influenced by the presence of glucose. The analysis of EPSs in terms of rheological properties showed a pseudo plastic behavior coupled to an elevated apparent viscosity, which increased with the EPS concentration, even if the viscosity value was constant in all the studied conditions of pH, temperature and salinity. This polysaccharide together with its producing microorganism could represent a valuable candidate for the recovery of oil, especially in oil deposits with high saline concentrations.

Another Archaea EPS producer is the heterotrophic facultative sulfur-dependent hyperthermophilic *Thermococcus litoralis* which was able to synthesize exopolymers in a sulfur-free defined medium with a growth performance similar to that registered on complex media [48]. This microorganism, isolated from a shallow submarine thermal spring, has an optimal temperature of growth of 88 °C. The exopolysaccharide from *T. litoralis* displayed an unusual structure since it was formed by mannose that normally is found as a monomer sugar in polysaccharides from plants or yeasts. Moreover, 1% to 2% sulfate and 1.5% to 4.5% phosphorus substituents were also found in *T. litoralis* EPS.

In the Bacteria Domain, some marine thermophilic bacilli species have been reported as EPS producers, for example the *Bacillus thermodenitrificans* strain B3-72. The *B. licheniformis* strain B3-15, isolated from hot marine shallow vents of Vulcano island (Italy), is able to produce interesting EPSs that show antiviral activities [49,50]. The *B*. *licheniformis* strain T4 isolated from the Panarea island (Italy) is able to produce a fructo-fucan polymer that has an anti-citotoxic activity [51]. *G. tepidamans* V264, isolated from a Velingrad Bulgarian hot spring, is able to produce an unusually thermostable glucan [52]. And finally, *Aeribacillus pallidus* 418, isolated from the Bulgarian Rupi hydrothermal springs, is responsible for the synthesis of two novel EPSs [53]. 

Lin *et al.* [54] characterized the primary structure of a novel exopolysaccharide TA-1 secreted by *Thermus aquaticus* YT-1 that shows immune modulatory effects in murine macrophage and in human monocyte cell lines. 

Among the extremophilic microorganisms, the halophiles represent the community that tolerates and/or requires high salt concentrations for their growth and that are widely demanded by several industrial sectors for their novel enzymatic activities and products with several possible applications [55]. In the *Haloferax*, *Halobacterium*, *Natronococcus*, *Halococcus*, and *Haloarcula* genera, many microorganisms are present that are able to produce EPSs. Considering the *Halomonas* genus, the species *H. maura*, *H. eurihalina*, *H. ventosae*, *H. anticariensis and H. alkaliantarctica* isolated from hypersaline environments, were shown to be good EPS producing species [56,57,58,59,60,61,62,63,64]*.* Typically the exopolysaccharides from *Halomonas* strains are characterized by a high presence of sulfate groups and uronic acids responsible for their gellifying actions. 

## 4. EPS Production and Recovery

The improvement of production relies on fermentation techniques involving new strategies like the use of genetically engineered microorganisms and new methodologies, which result in high yield and cost-effective production of these biopolymers. The commercial exploitation of the biochemical microbial diversity has resulted in the development of the fermentation industry. Moreover, the new techniques of genetic manipulation have given the opportunity to develop new processes and to improve old ones [65]. The recovery of EPS, *i.e.*, the “downstream processing”, requires the classical steps of the recovery of the product from the fermentation broth after the removal of insolubles (for example cells) by centrifugation, followed by isolation and purification steps. However, the EPS-producing cultures represent a challenge for the culture producers because of the high viscosity of the fermented growth media that makes the separation of the cells by centrifugation very difficult, which in turn results in serious problems related to heat transfer and oxygen supply [65]. In this case, a fed-batch culture is suitable to bypass highly viscous broths, even, as in the case of thermophilic EPS producers, the high temperature utilized for the growth of microorganisms can solve the problem.

At the end of the fermentation processes, the EPS can be recovered from the growth medium by separating the cells by centrifugation. Subsequently, the exopolysaccharides present in the cell-free medium are precipitated using ethanol or acetone or methanol. In a next step, the pellets that are recovered by centrifugation, go on to appropriate dialysis in distilled water, and are then freeze dried in order to achieve the crude EPS.

The limit of bacterial EPSs for industrial use can be ascribed to the scarce extent of their yields even if there are some strains of *Xanthomonas campestris*, *Bacillus polymyxa*, *Klebsiella pneumonie* and *Sfingomonas elodea*, for example, that are known for their high levels of biopolymer production [66,67,68,69,70].

Since the microbial EPSs possess different sugar compositions and different rheological characteristics, they represent a very big potential reserve within which it is possible to find the ideal EPS to use in a specific biotechnological application. 

## 5. Fermentation Strategies

### 5.1. Effect of the Chemical Composition of the Medium and the Physical Parameters of Growth Conditions

The exopolysaccharide yields and the rheological aspects of these polymers depend on several critical factors related to fermentation conditions: carbon and nitrogen source utilization, mineral requirements, temperature and optimal pH but also agitation rate and oxygen consumption, just to give some examples (Table 1). From the literature, it is possible to assert that the nutritional and environmental conditions (culture conditions) can affect the yield and quality of microbial exopolysaccharides, whereas the prediction of molecular mass, number of monosaccharide units and degree of branching of EPS is possible by using a physiological control [9]. The nutrient limitation in marine bacteria (such as nitrogen, phosphorus, sulfur and potassium) can cause an increase in EPS production as stated by Sutherland [16]. Moreover, by setting several physical stressing factors, such as temperature, osmotic stress or others responsible for decreased growth, the EPS production may be enhanced. Furthermore, the selection of a most suitable carbon source for the growth medium composition represents the right effort to optimize the polymer production [23].

In recent times, Marx *et al.* [71] investigated the influence of temperature, salinity and pressure on EPS production in the psychrophilic gamma-proteobacterium *Colwellia psychrerythraea* strain 34H isolated from Arctic marine sediments. The authors noticed that the stressful environmental conditions increased EPS production; indeed in a range of temperatures between −8 °C to −14 °C, the EPS yield rose significantly. Similarly, higher pressures (400–600 atm) as well as higher salinity (10–100 parts per million, at the temperatures of −1 °C and −5 °C) significantly improved the production of EPS. 

Mancuso *et al.* [72] described the effects of incubation temperature on growth and production of EPS by Antarctic sea ice bacterium grown in batch culture. In particular, when the strain CAM025 (member of the genus *Pseudoalteromonas*) was grown at −2 °C and 10 °C, the yields of EPS were 30 times higher than those at 20 °C, confirming the hypothesis of the cryoprotective role of EPS in the case of extremely low temperatures. Moreover, the EPS produced at −2 °C and 10 °C had a higher uronic acid content than that produced at 20 °C. 

The effect of the growth temperature on the production of an exopolysaccharide named EPS-R [73] by the marine microorganism *Hahella chejuensis*, collected in Cheju Island, was studied as well. The highest productivity was obtained in the 20 to 25 °C temperature range while above 30 °C, productivity was reduced. In this study, other parameters were considered in order to optimize the production of EPS: In particular the authors noticed that among the nitrogen sources tested, tryptone provided the highest production level while among the carbon sources, sucrose was the best. In both cases, the best productivity (EPS/dried cell weight) was achieved with a sucrose/tryptone ratio of 2:1. The effects of NaCl, MgSO_4_ and CaCl_2_ on EPS production was also noticeable: These salts that were all required for the growth of this microorganism caused the highest accumulation of polymers when they were added to the media at 1%, 0.5% and 0.01% w/w, respectively.

Al-Nahas *et al.* [74] described the characterization of an EPS producing bacterium, named *Pseudoalteromonas* sp. AM, isolated from a sponge sample at about 16 m depth in the Red Sea (Hurghada, Egypt). The production of this EPS was achieved by fermentation in 250 mL shake flasks containing 50 mL of casein hydrolysate-glucose broth at 30 °C. The effect of different carbon sources including galactose, fructose, lactose, sucrose, dextrin, glycerol, molasses and citric acid, was tested. In addition, different nitrogen sources such as meat extract, yeast extract, tryptone, peptone, ammonium sulfate, ammonium oxalate and sodium nitrate were also tested. The results showed that the maximum EPS productivity was achieved after 7 days at pH 7, at an agitation of 150 rpm in a fermentation medium containing glucose, meat extract and 3% NaCl.

Recently, a novel bacterium strain BM39, affiliated with *Pantoea* sp., isolated from sediments collected in the Tyrrhenian Sea, was selected for its ability to produce very high levels of glucan EPS [66]. Kinetic studies of EPS production by strain BM39 cultivated in shaken cultures on different media indicated that the maximum EPS production (21.30 ± 2.03 g/L) was obtained in EMG medium (containing glucose 80 g/L as carbon source) with a maximum of EPS time productivity of 0.89 ± 0.09 g/Lh; using a medium containing 80 g/L of sucrose (EMS medium) or 80.0 g/L of fructose (EMF medium) as carbon sources, the EPS productions registered were 11.82 ± 1.06 g/L and 11.05 ± 1.17 g/L, respectively, while the productivity was 0.39 ± 0.04 g/Lh and 0.37 ± 0.04 g/Lh, respectively.

The effect of carbon sources was also studied in *Zunongwangia profunda* SM-A87: The maximum EPS production, about 6.47 g/L, was detected when lactose was added to the basic marine medium with the other carbon sources tested being glucose, mannose, maltose and sucrose [75].

The polysaccharide 1644 produced by a strain of *Alteromonas* sp. showed a unique rheological behavior [76]. The purified EPS, in the presence of divalent ions, formed an elastic gel, which can be stretched reversibly without any loss of structure [36]. Dubreucq *et al*. [77] clarified its structure indicating the presence of an unusual dicarboxylic acid sugar in the repeating unit, probably responsible for the high affinity of this polymer for divalent ions. In contrast to some other bacterial EPSs, which are synthesized during active bacterial growth, the polysaccharide 1644 is produced only during the initial exponential growth phase. In order to improve its production, Saiman *et al.* [76] checked the use of a mineral-defined medium containing ammonium chloride as sole nitrogen source instead of peptone and yeast extract. The fermentation strategy started with an ammonium chloride concentration of 0.4 g/L. When the microorganism reached the initial exponential phase, the ammonium chloride level was kept continually low, but the concentration was enough in order to permit protein synthesis. The synthesis of EPS was continuous during the nitrogen source use and persisted linearly up to the conclusion of the fermentation, generating EPS yields 50% higher than those obtained with the complex medium.

Llamas *et al.* [31] studied the heteropolysaccharide produced by the strain of *Salipiger mucosus* A3^T^, a halophilic species belonging to the *Alphaproteobacteria* genus and isolated from the Spanish Mediterranean seaboard. The kinetics of its production illustrated that the polymer was synthesized essentially during the exponential phase but persisted during the stationary phase with a lower yield. EPS was excreted mainly during the exponential growth phase but continued to a lesser extent during the stationary phase reaching the value of 120 mg EPS/100 mL broth. Moreover, EPS yield was always related to the quantity of biomass in culture and the EPS production was best at a 2.5% salt concentration. As far as incubation temperature was concerned, EPS and cell growth yields were lower at both 22 °C and 42 °C than at 32 °C. Similar results were achieved when the pH value of the medium was lower or higher than 7.0. The same trend was also obtained for both static incubation or at a stirring rate of more than 100 rpm, causing comparably lower growth and lower EPS yields [31].

In the hyperthermophilic *Thermococcus litoralis*, the EPS production was significantly stimulated by adding NH_4_Cl in continuous culture at high dilution rates: The EPS, acting as a transporter of reducing equivalents out of the cell, allows the microorganism’s survival [78]. Another Archaea that secretes exopolysaccharides as a defense mechanism when exposed to stress is *Archaeglobus fulgidus*, an anaerobic marine hyperthermophile [79]. Studies of biofilm production showed that the biofilm secretion was stimulated by exposure of the microorganism to stress factors, such as pH and temperature, or high concentrations of antimicrobial agents. 

The effect of carbon sources was also studied in *Aeribacillus pallidus* 418 [53] in which a basal medium supplemented with maltose (0.9%) gave the best EPS and biomass production, of 80 μg/mL and 0.75 mg/mL, respectively. Moreover, regarding the nitrogen source, the highest EPS production was observed when ammonium salts (0.2%) were added to the basal medium, while organic sources provided a better growth. Regarding the effects of temperature (between 50 and 70 °C with 5 °C temperature steps) and pH value (between 6.0 and 8.5 with 0.5 pH steps) on EPS synthesis, the highest polymer production was observed at 55 °C and pH 7.0.

As reported until now, the abundance and the structure of polysaccharides are mainly affected by the culture medium composition. Usually sugars are found as the preferred carbon source to synthesize such exopolymers. Indeed, the haloalkalophilic *Halomonas alkaliantarctica* strain CRSS produces two different exopolysaccharides, a mannan or a xylo-mannan type glycan, when cells are grown on two different complex media, while a different polysaccharide with higher yield, a fructo-glucan type, is released in the presence of sodium acetate as sole carbon source in the minimal medium [64]. Additionally, thermophilic bacteria showing mucous colonies and belonging to *Bacillus-Thermus* genera have been isolated from shallow, marine hydrothermal vents of the flegrean area in Italy and have been characterized as EPS producers [80]. Among the different strains isolated, the strain 4009 collected in Ischia (Sorceto) island, reached an EPS yield of 60 mg/L using trehalose as sole carbon source, a value that was about 1000-fold compared to the minimal medium. 

**Table 1:** Examples of fermentation strategies for EPS production in marine microorganisms.

Microorganisms	Source	Max EPS-production	EPS Fermentation-increasing strategies	Production increment (fold)	References
*Aeribacillus pallidus* 418	Hot springs Bulgaria	0.13 g/L	Carbon and nitrogen sources: maltose, NH_4_Cl; temperature	2	Radchenkova *et al.*, 2013 [53]
*Alteromonas* sp. 1644	Hydrothermal vents East Pacific Rise	7.5 g/L	Nitrogen source: ammonium chloride	1.5	Samain *et al.*, 1997 [76]
*Hahella chejuensis*	Cheju Island	9.23 g/L	Carbon/nitrogen ratio: sucrose, tryptone; temperature, pH	n.r.	Sung-Hwan Ko *et al.*, 2000 [73]
*Halomonas alkaliantarctica* strain CRSS	Cape Russell lake, Antarctica	2.9 g/g dry cells	Carbon source: maltose	6	Poli *et al.*, 2004 [64]
*Pantoea* strain BM39	Sediments in Tyrrhenian Sea	21.30 g/L	Carbon source: glucose	2	Silvi *et al.*, 2013 [66]
*Pseudoalteromonas* sp. AM	Sponge sample in Red Sea	10.51 g/L	Carbon and nitrogen sources: meat extract, glucose; NaCl; pH; agitation speed	5	Al-Nahas *et al.*, 2011 [74]
*Pseudoalteromonas* strain CAM025	Antarctic sea ice	99.9 mg/g dry cells	Temperature	30	Mancuso *et al*., 2005 [72]
*Salipiger mucosus* A3^T^	Spanish Mediterranean seaboard	1.2 g/L	Stirring rate; incubation temperature; pH	n.r.	Llamas *et al.*, (2010) [31]
Thermophilic bacterium strain 4009	Ischia (Sorceto) Island	60 mg/L	Carbon sources: trehalose	1000	Nicolaus *et al.*, 2002 [80]
*Zunongwangia profunda* SM-A87	Deep-sea sediment southern Okinawa Trough	8.90 g/L	Carbon and nitrogen sources: lactose, peptone; temperature; statistical approach	10	Liu *et al*., 2011 [75]

n.r.: Not reported.

### 5.2. Alternative Cheap Sources for EPS Production

Most of the costs related to the achievement of EPSs in industrial processes are due to the media composition. Sugars are the common substrates utilized during the microbial fermentation to produce EPSs. Nowadays, there are several reports that describe the formation of exopolymers using alternative and cheaper carbon sources, such as industrial residual materials, agro wastes, *etc*. This new slant, if producing good results, is to be preferred, because it represents an opportunity to produce valuable substances like exopolysaccharides with a wide range of biotechnological applications using cheaper substrates, and at the same time to contribute to waste disposal, which is an expensive process in itself. 

More *et al.* [81] described the isolation from wastewater sludge of thirteen EPS-producing bacterial strains that belong to the *Bacillus*, *Serratia* and *Yersina* genera*.* All the isolated strains showed the ability to synthesize extracellular polymeric substances, even when using secondary wastewater sludge (without chemical polymers) as the growth media. 

For the soil isolate *Bacillus subtilis*, agro substrates, cane molasses, and rice bran in different concentrations were tested to enhance the EPS production with respect to sucrose; cane molasses at a concentration of 2% lead to the highest yield of 4.86 g/L compared to a medium with sucrose (2.98 g/L) as sole carbon source [82]. Küçükaşik and co-workers [83] also tested pretreated sugar beet molasses and starch molasses as carbon sources for levan production by the *Halomonas smyrniensis* strain AAD6, and both substrates resulted in working alternatives to sucrose. In particular, when using 50 g/L of sucrose in the growth medium, the levan yield was 1.84 g/L, whereas in the presence of 30 g/L of sugar beet molasses as sole carbon source, the achieved levan production was 12.4 g/L. The soil isolate levan producer *Bacillus polymyxa* (NRRL-18475) was found to be able to synthesize the EPS using appropriately modified sugarcane juice and beet molasses with a satisfactory yield [84]. A molasses-based medium has been demonstrated to also be suitable for gellan gum production by *Sphingomonas paucimobilis* ATCC-31461 [85]. Sugar beet molasses used as sole carbon source was also shown to be suitable for xanthan gum fermentation by *Xanthomonas campestris* ATCC 1395 [86].

In the literature, there is no study concerning the improvement of the EPS production process by marine bacteria; nevertheless it would also be interesting to investigate the potentiality of these microorganisms to secrete EPS utilizing alternative carbon sources.

### 5.3. Statistical Tools

The microbial exopolysaccharides are fermentation products the yield, composition and, therefore, properties of which are the result of the combination of numerous variables, as explained in this review. Clearly, to reach the best fermentative conditions, several experiments must be done and several hypotheses must be developed, without any outcome warranty and with expenditure of chemicals, time and energy. Increasingly, statistical approaches are also being propagated in the field of EPS production. Their use provides an immediate instrument to individuate the suitable parameters to be modified and to decide in which direction to go in order to obtain with high probability what research is interested in. This way, the number of experiments can be drastically reduced, as they are more specifically focused. In the literature, Plackett-Burman (PB) design and response surface methodologies (RSM) are the most common approaches reported for the optimization of the EPS production process. The PB experimental design [87] is a valuable tool generally used to screen main factors from a number of process variables. The RSM is an efficient statistical strategy widely selected to improve product yields and to reduce overall process time and costs; it is suitable for planning experiments and predicting the optimal conditions of parameters involved in a process [75]. Qiang *et al*. [88] used RSM for EPS production by *Klebsiella* sp. H-207, isolated from activated sludge. A maximum polysaccharide yield of about 15.05 g/L was achieved under optimized conditions in terms of both medium composition and culture conditions, which consisted of sucrose 31.93 g/L, KNO_3_ 2.17 g/L and K_2_HPO_4_ 5.47 g/L, a seed age of 13 h, with an inoculum size of 10.6% and an incubation temperature of 28.9 °C. *Zunongwangia profunda* SM-A87 isolated from deep-sea sediment, is the first marine bacterium that produces EPS with the high yield of 8.90 g/L. On the basis of this result there is an RSM approach that is used to select the more sensitive variables with respect to the study objective and results in the necessary amount of lactose and peptone and the incubation time [75].

Screening of the most significant fermentation parameters affecting levan production was done by the PB design for the microbial strain *Pseudomonas fluorescens* NCIM 2059. Six nutritional variables (sucrose, casein peptone, NH_4_Cl, KH_2_PO_4_, MgSO_4_, and NaNO_3_) were studied to define the optimal medium. A significant increase of levan yield from 5.27 up to 15.42 g/L under these conditions was found [89]. 

### 5.4. Genetic Approach

The exopolysaccharide production is a genetically determined process. Hence, in order to improve the microbial EPS synthesis, an accurate approach should first identify all the genes responsible for polymer synthesis and then try to comprehend the mechanisms involved. This represents what science has been doing in the last decade, giving more and more privilege to studies on a genomic level. 

Effectively, once a complete view of a microbial EPS-producing genome has been obtained, it is possible to choose the right strategy to improve the polymer release through manipulation of the genes encoding the enzymes involved in the biosynthesis, or by altering the regulatory pathways that affect gene expression and enzyme activity, as well as by selecting the most suitable substrate to be supplied to the microorganism during growth. 

To our knowledge, in the field of marine EPS-producing bacteria, the performed genomic studies have provided clear indications only regarding the adaptation and evolution mechanisms needed to survive in determinate environmental growth conditions, hence attributing a relevant ecological role to the exopolysaccharides [30].

*Zunongwangia profunda* SM-A87, as many other deep-sea isolates, produces large quantities of exopolysaccharide [75]. Its genome was the first sequenced in the phylum of *Bacteroidetes* [90]. Apart from various extracellular enzymes for carbohydrate, lipid and DNA degradation, it possesses two polysaccharide biosynthesis gene clusters. Its genome analysis reveals the presence of some common features of deep-sea bacteria.

The deep-sea psychrotolerant gammaproteobacterium *Pseudoalteromonas* sp. SM9913 also produces large quantities of EPS [28]. This EPS was able to enhance the stability of the cold-adapted protease MCP-01 secreted by the same strain through preventing its autolysis. In addition, an ecological role connected to the adaptation to the deep-sea ecosystem was attributed to the purified EPS. The *Pseudoalteromonas* sp. SM9913 genome was sequenced, and a comparative study with the closely related Antarctic surface sea-water ecotype *Pseudoalteromonas haloplanktis* TAC125 (TAC125) was performed by Qin *et al*. [91]. Some deep-sea bacteria the genomes of which have been sequenced, such as *Idiomarina loihiensis* [92] and *Alteromonas macleodii* “deep ecotype” [93], present EPS biosynthesis genes, thus confirming that the production of EPS may be a common strategy that deep-sea bacteria adopt to endure extreme conditions. 

Even if, to our knowledge, there are no cases of marine bacteria whose genome sequence analysis has been used to increase the exopolysaccharide yield, it would be desirable to extend this approach to these microorganisms in the near future. 

On the contrary, in the case of the halophilic *Halomonas smyrniensis* strain AAD6 levan producer, the knowledge of the genome reconstruction (first performed using the microorganisms *Chromohalobacter salexigens* DSM 3043 that is taxonomically close to *H.* *smyrniensis* [21], and then from the whole-genome sequence) allowed the prediction of the stimulatory effect of mannitol on levan biosynthesis, which was later experimentally confirmed by Ates *et al.* [22]. Several other metabolic engineering efforts to improving production of sugar polymers have been attempted; for instance for the production of the following EPS: xanthan [94], gellan [23], cellulose [95] and levan [94]. Not all attempts have been successful; indeed only for cellulose was an increase polymer yield obtained [94,95]. Unfortunately, in the other cases, the results were not as encouraging, most probably because it would be necessary to get a deeper insight into both the single steps and the overall regulation of the pathways [23,94].

## 6. Conclusions

**Figure 1:**
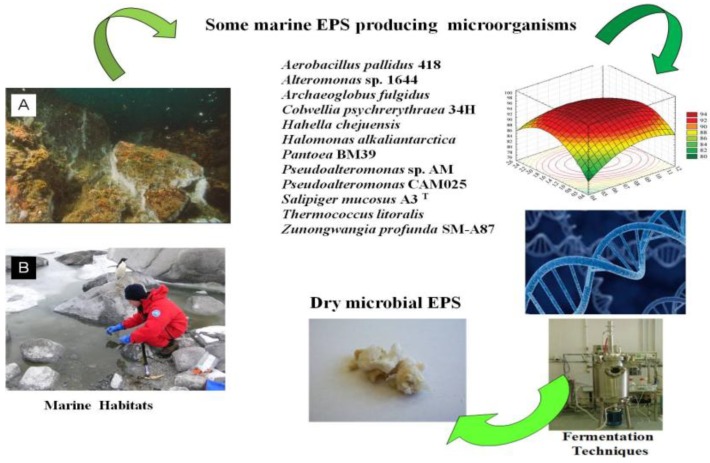
Examples of marine exopolysaccharides (EPS) producers and improvement strategies for EPS production. (**A**) “Secca Fumosa” located in the Gulf of Pozzuoli, Naples, Italy, taken by Dr. Guido Villani; (**B**) Samples collected by Dr. Annarita Poli in the “Cape Russell” lake in the Ross Sea, Antarctica (74 52.35 S 163 53.03 E) during the XXI Italian Antarctica Expedition.

Although diverse microbial exopolysaccharides have been studied in the last years, only little information is available regarding their hyperproduction. In order to improve the fermentation processes of bacterial EPS, it is possible to operate at different levels, by searching for the ideal nutritional conditions resulting in the greatest yield of EPS with more suitable biotechnological proprieties, as well as by operating at the genomic level by inserting or finding those genes that are directly involved in sugar metabolism (Figure 1). Both strategies, possibly also applied together, can lead to promising results as a consequence of an interdisciplinary approach based on the analysis of complex system biological interactions and supported by statistical tools and predictive studies.
